# Distribution of wealth quintiles and risk factors of non-communicable diseases in Ghana: evidence from the Ghana demographic and health survey 2014 using concentration curves model

**DOI:** 10.11604/pamj.2021.40.262.31579

**Published:** 2021-12-23

**Authors:** Brenyah Joseph Kwasi

**Affiliations:** Department of Global and International Health, School of Public Health, Kwame Nkrumah University of Science and Technology, Kumasi, Ghana

**Keywords:** Distribution, wealth quintiles, risk factors of non-communicable diseases, Ghana, demographic and health survey, concentration curve

## Abstract

**Introduction:**

in recent times, the assertion of non-communicable diseases afflicting the rich has been demystified but cuts across the rich and the poor. Individuals in all categories of wealth quintiles are affected by the risk factors of non-communicable diseases such as alcohol consumption, tobacco use, unhealthy dietary practices and physical inactivity. However, information on the distribution of these risk factors across different socio-economic status is scanty. This study assessed the distribution of wealth quintiles and the risk factors of non-communicable diseases, using the concentration curve model.

**Methods:**

it was a quantitative study with analytical design using the Ghana Demographic and Health Survey (GDHS), 2014 data. The variables of interest were income status of respondents and risk factors of non-communicable diseases. In the analysis, income levels were categorized into wealth quintiles with assigned percentages (25%, 50%, 75% and 100%) denoting poor, rich, richer and richest respectively. The risk factors of non-communicable diseases were also categorized and assigned percentages (relatively exposed 25%, exposed 50%, more exposed 75% and most exposed 100%). A concentration table was employed to assess the risk factors of non-communicable diseases labelled X-axis and wealth quintiles labelled Y-axis. The cumulative percentage of the wealth quintiles (Y-axis) were plotted against the cumulative percentage of the risk factors of non-communicable diseases on the X-axis.

**Results:**

the study found moderate concentration of alcohol consumption among the middle to upper wealth quintiles (richest). Again, the study revealed that, wealth quintiles are practically indifferent to tobacco use and that both the rich and poor equally and minimally use tobacco as the concentration curve is very close to the perfect line of equality (45°). This study found near equal distribution of unhealthy dietary practices among the rich and poor in Ghana. It was found that, 40% - 80% of rich people were physically inactive with the application of a physical activity level of 100%. It was noticed that, 40% of the rich people only performed 20% of physical activities.

**Conclusion:**

the study concludes that; wealth quintiles have implications for the risk factors of non-communicable diseases.

## Introduction

Decades ago, non-communicable diseases (NCDs) such as diabetes, strokes, hypertension and cancers were considered diseases of the rich and elderly [1,2]. Not only are NCDs more pronounced and affecting more than half of people in low-and middle-income countries (LMICs) but also responsible for more than 79% of global illness and 30% of deaths under the age 60 [3,4]. While a lot has been reported about the death tolls from NCDs, very scanty information is available on the distribution of occurrence of NCDs within the population across different socio-economic groups. This study sets to investigate the distribution of wealth quintiles and risk factors of non-communicable diseases in Ghana based on evidence from the Ghana Demographic and Health Survey 2014 using concentration curves model. In 2010, out of the 52.8 million deaths worldwide, 34.5 million were due to NCDs and 80% of these deaths were in low-and middle-income countries [4, 5]. Global policy experts predicted that, by 2020, NCD cases may cause seven (7) out of ten (10) deaths in developing countries [6]. The sub-Saharan Africa context is not different from the global NCDs patterns, distribution, health inequalities and disabilities. Available records in Ghana revealed that, hypertension, stroke, diabetes and cancers are among the top 10 causes of hospital admissions and deaths [7-10]. While the occurrence of NCDs have biological and genetic disposition, the neglect of some modifiable behaviours, social, economic and political factors have also manifested as key accentuating factors which have implication for the occurrence of NCDs. For instance, modifiable behaviours such as excessive alcohol intake, excessive tobacco use, unhealthy dietary practice and physical inactivity play out as major risk factors of NCDs [6,11,12]. With the onset of rapid epidemiological transition [2], the risk factors of NCDs are accentuated by socio-economic and political influences affecting both rich and poor with the incidence falling severely on the poor especially, those in developing economies including sub-Saharan Africa [13,14]

Therefore, vulnerable and socially disadvantaged people acquire NCDs more and die sooner than people of higher social positions such as the more educated, people with good occupation, high income earners and among others [15]. There is therefore the evidence that, social and economic status of a person and NCDs occurrence are associated [16,17]. However, social and economic status in low and middle-income-countries (LMICs) just like high-income countries are in gradients. Within the low-income countries, not much attention has been paid to how the people in the various wealth quintiles are distributed in terms of exposure to the risk factors of NCDs. In many countries, research outcomes have revealed that, lowest income households are associated with high level of NCD risk factors due to lifestyles. For instance, in Nepal, the poor spend about 10% of their income on cigarettes whiles Romanians and Zimbabweans spend 11% and 7% of their income on alcohol consumption [18]. Again, unhealthy dietary practices are also linked to NCDs occurrence. Consumption of processed and fast foods have increased in LMICs. This nutritional transition has influenced dietary behaviour within the population and increased the risk of acquiring NCDs. Consumption of plant-based diets such as fruits and vegetables reduce obesity, diabetes and cardiovascular diseases. However, very few people in LMICs are able to afford these healthy products [6]. The above narration have given broad overview of the magnitude and devastating nature of NCDs. The outcome of this study would promote continuous exchange of emerging ideas on NCDs occurrence, pattern, distribution and burden across different socio-economic groups. These may impact on NCD policy makers and partners to strategize for formidable polices on NCDs in Ghana. Studies relating to wealth quintiles which influence categorization of socio-economic groups demand nationally validated data and the GDHS, 2014 becomes appropriate. This paper examines the distribution of wealth quintiles with risk factors of NCDs using the Ghana Demographic and Health Survey 2014 data by employing the concentration curve model.

## Methods

The study was an analytical design which employed the quantitative approach to address the objective by using the secondary data of the Ghana Demographic and Health Survey (GDHS), 2014.

**Study setting:** proposals, selection of study sites and other documentations started in 2016 before the creation of additional regions in Ghana. The study was therefore done in 4 out of the 10 regions which existed in Ghana. These were Ashanti Region, Brong Ahafo Region, Northern Region and Greater Accra Region. The study was conducted between 2018 to 2019.

**Study variables:** the variables of interest extracted from the GDHS were income status of respondents, risk factors of NCDs such as alcohol intakes, tobacco use, unhealthy dietary habits, physical inactivity and income status. All these variables were recoded. There was categorization of the income into wealth quintiles against the risk factors of NCDs with assigned percentages. The income levels were therefore pulled and categorized into poor, rich, richer and richest. Since, the categories were 4 and the summation has to be 100%, each category was relatively assigned 25% (25%, 50%, 75% and 100%) denoting poor, rich, richer and richest respectively. The risk factors of NCDs based on the responses of respondents were also pulled, categorized and assigned percentages such as relatively exposed to NCDs (25%), exposed to NCDs (50%), more exposed to NCDs (75%) and most exposed to NCDs (100%). The was done for all the four (4) identified risk factors of NCDs (alcohol intake, tobacco use, unhealthy dietary behaviour and physical inactivity).

**Bias:** since the data was developed by the Ghana Statistical Services and nationally validated, the tendency of biases had been catered for already. This study therefore selected the required variables and recoded.

**Study size:** the GDHS is a nationally representative survey of 9,396 women and 4,388 men from 11,835 interviewed households. This sample was filtered using age limit of 30 years and above, NCDs status of respondents, risk factors of NCDs (physical inactivity, alcohol consumption, tobacco consumption, unhealthy dietary practices) and income status to get a sample of 4,122 which was used for the study.

**How quantitative variables were handled:** concentration table was formulated (Table 1). The concentration table provided a means of assessing the degree of inequality in the distribution of occurrences. Two key variables underlie the concentration table formulation. First, the set of variables which are the risk factors of NCDs (risk factors against which the wealth quintile distribution is to be assessed) and labelled X-axis and secondly, the wealth quintiles which are the variables of distribution which are the subject of interest) and labelled Y-axis as shown below. Again, of a particular variable X which can be alcohol consumption, the sub-categories were (relatively exposed, exposed, more exposed and most exposed). Similarly, for a variable like wealth quintiles, the sub-categories were poor, rich, richer and richest. Each of the risk factors were operated with the wealth quintiles to formulate four sets of concentration tables. The format for the development of one such table is demonstrated on Table 1 (wealth quintiles and alcohol intake variables). Similar tables were made for wealth quintiles and unhealthy dietary practice, wealth quintiles and tobacco use and lastly, wealth quintiles and physical inactivity (Table 1). The next level of analysis was the use of the concentration curve. The concentration table was then plotted on a graph. The cumulative percentage of the wealth quintiles variables (y-axis) were plotted against the cumulative percentage of the NCDs risk factors variables, beginning with the least significant level of exposure to NCDs risk factor to the significantly most exposed NCDs risk factor (X-axis). The researcher plotted the cumulative % X twice on the graph for X-axis and once on Y-axis to obtain the 45° line of perfect equality in the distribution. To get the concentration curve, the researcher again plotted the same cumulative % of X for X-axis and then plotted the cumulative %Y for the Y-axis. The interpretation of the nature of relationship between the perfect line of equality (45°) and the concentration curve is that, the closer the concentration curve to the line of equality, the lesser the concentration of the wealth quintiles to the risk factor of NCD under consideration. This means that, the wealth quintiles are not distributed nor exposed or are less exposed to a risk factor of NCDs.

**Table 1:** wealth quintiles and risk factors of non-communicable diseases (alcohol intake, unhealthy dietary practices, physical inactivity, tobacco consumption)

Variable	Frequency	%	Cum %	Variable	Frequency	%	Cum %
X	X	X	X	Y	Y	Y	Y

**Ethical considerations:** the study followed all the ethical considerations in research. The Ghana Demographic and Health Survey (GDHS) is a secondary data of Ghana. It was conducted by the Ghana statistical service. The raw data is made available to all Institutions and my institution readily have it. We use this data for an aspect of our advanced quantitative analysis course. Ethically, I cleaned the data, recoded the variables of interest to my specification and generated results.

**Figure 1:**
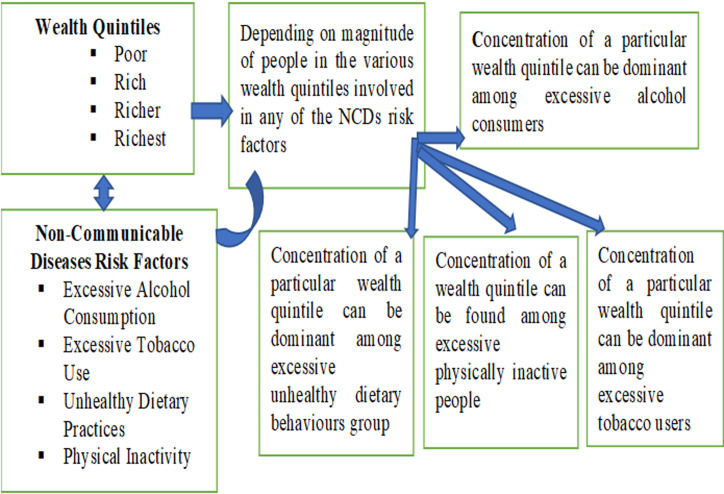
concept of distribution of wealth quintiles among risk factors of non-communicable diseases

**Conceptual framework:** the study was conceptualised based on wealth quintiles and the magnitude of such persons in the respective wealth quintiles exposed to a particular risk factor of NCDs such as alcohol intake, tobacco use, unhealthy dietary practice and physical inactivity as shown on Figure 1. Wealth status may influence the modifiable risk factors of NCDs such as alcohol intakes, tobacco use, dietary practices and physical inactivity. The level of influence may depend on the number of people from the various wealth quintiles (Y) who are directly involved in the practice of any of the risk factors of NCDs (X) and that gives distributional effects establishing the relations between Y and X. If more of any particular wealth quintile or more of the various wealth quintiles are involved, there may be wider distribution and vice versa.

## Results

The concentration curve is used to show the distribution of a phenomena within an area. The concentration curve shows good visual impression and comparison of the observe differences from the co-efficient using the line of perfect equality of 45° angle. The more diverse and unevenly the sample spreads out, the wider the concentration. If the concentration curve is distant from the line of perfect equality, it means wealth quintiles have less effect on the NCDs risk factor under consideration.

**Figure 2:**
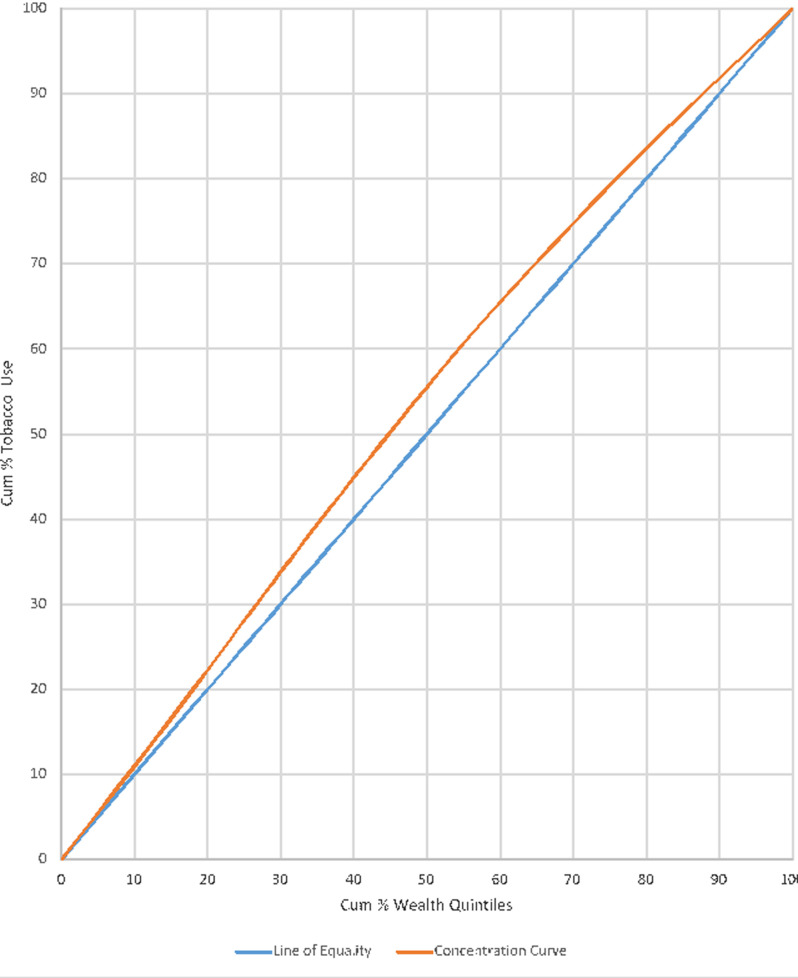
concentration of wealth quintiles and alcohol consumption

**Concentration of wealth quintiles and alcohol consumption:** in the GDHS, (2014), 4,077 households were involved in the interviews relating to alcohol consumption. Figure 2 reveals that, even though the concentration curve was quite close to the line of perfect equality of 45° angle, there was a bit of high concentration of alcohol consumption among the middle to upper wealth quintiles (25%-80% cumulative wealth quintiles).

**Figure 3:**
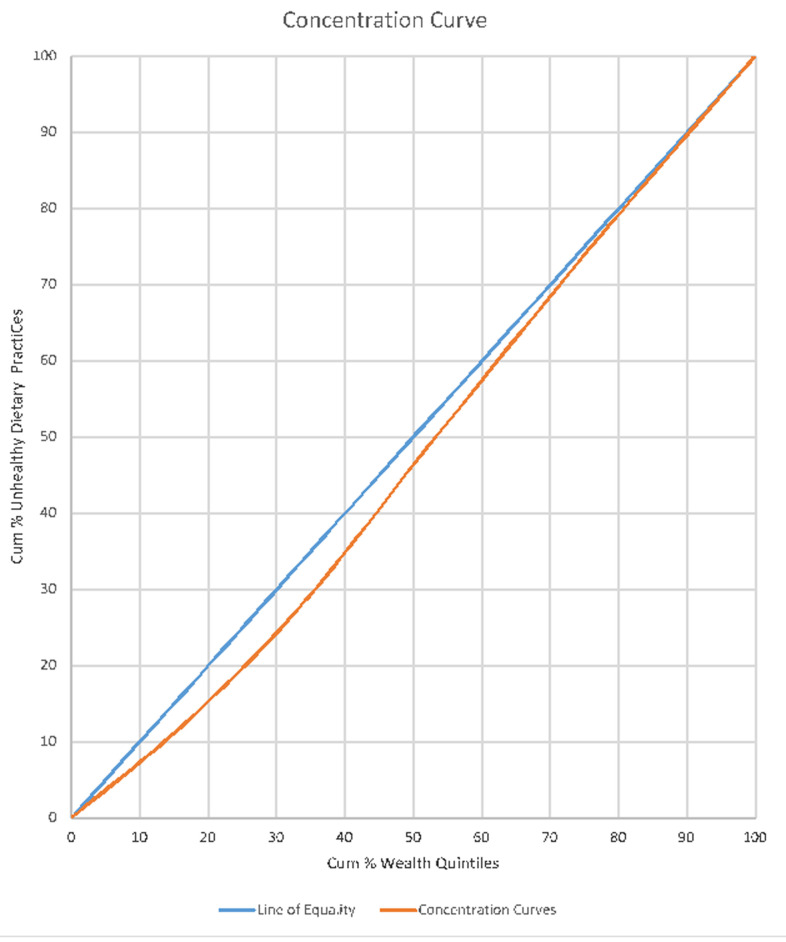
concentration curve of wealth quintiles and tobacco use

**Concentration of wealth quintiles and tobacco use:** the study found that, tobacco use is distributed equally among the wealth quintiles as the concentration curve is virtually equal to the 45° line of perfect equality (blue line). The implication is that, differences in wealth quintiles are almost practically indifferent to tobacco use and that both the rich and poor equally and minimally use tobacco as shown on Figure 3.

**Figure 4:**
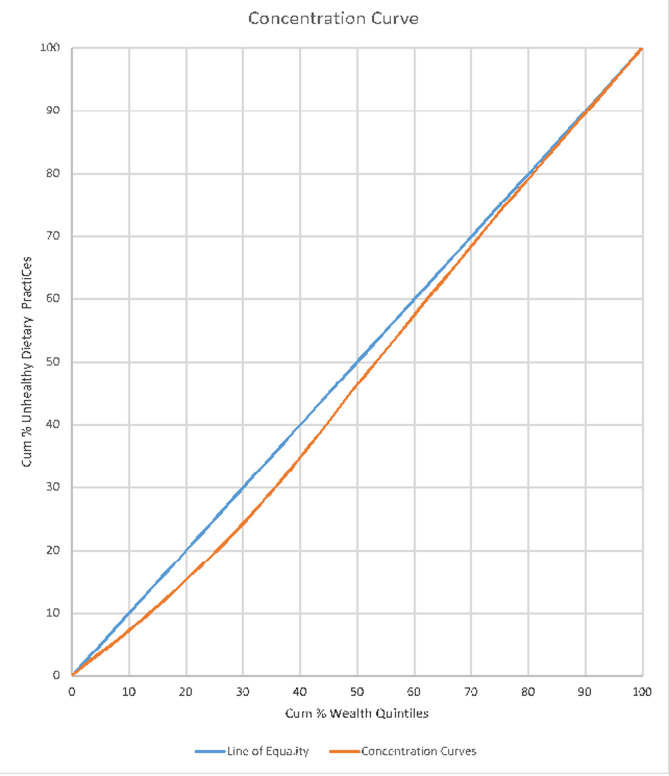
concentration curve of wealth quintiles and dietary practices

**Concentration of wealth quintiles and unhealthy dietary practices:** wealth status were applied on dietary practices. The study found that, the concentration curve is closely aligned with the perfect line of equality. This means there is near equal distribution of unhealthy dietary practices among the poor and rich people in Ghana. However, a closer look at the graph portrays that, the incidence of unhealthy dietary practice falls a bit on the less rich people who are within the (10% - 60% cumulative wealth quintiles bracket) as shown on Figure 4.

**Figure 5:**
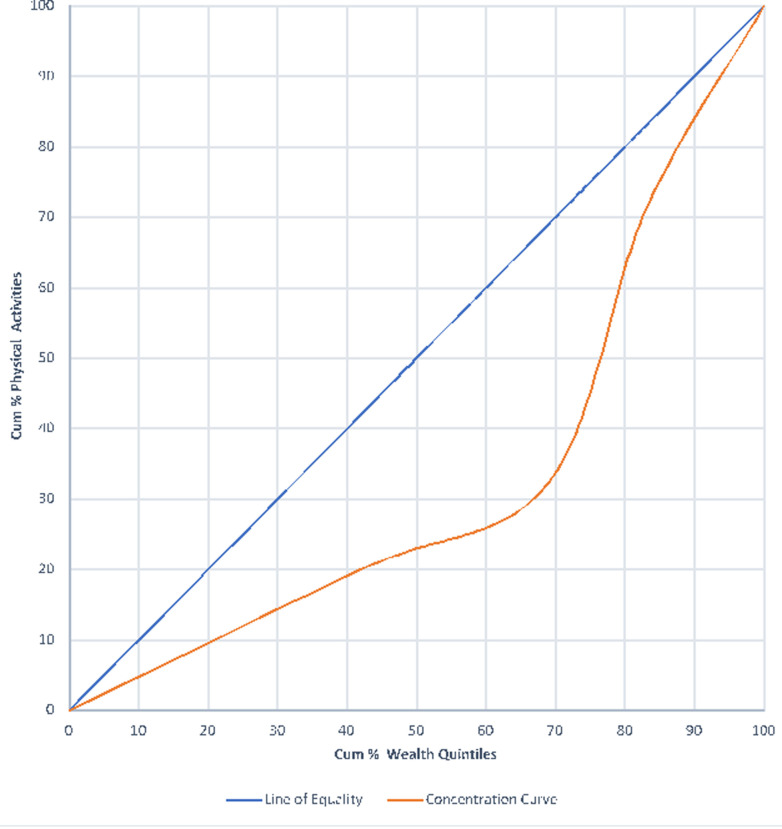
concentration curve of differences in wealth quintiles and physical activity

**Concentration of wealth quintiles and physical inactivity:** the study investigated the degree of concentration of wealth quintiles and physical inactivity using the GDHS 2014 data. It was found that, many people are physically inactive among the various wealth quintiles (Figure 5). About 40% - 80% of rich people were physically inactive with the application of a physical activity level of 100%. It was noticed that, 40% of the rich people only performed 20% of physical activities. Again, 80% of them performed about 60% of physical activities. The implication is that, physical inactivity is disproportionately concentrated among people of higher wealth quintile as illustrated on Figure 5.

## Discussion

The study outcomes have revealed that, poor people consume much alcohol as compared to the rich [19,20]. The concentration curve emerging out of the GDHS, 2014 national data corroborated this assertion and further revealed that, individuals in the richest (75% - 100% cumulative wealth quintiles bracket) are also not involved much in alcohol intake. The current study rather however found that, the incidence of alcohol consumption relatively falls on the rich (individuals in the 25% - 50% cumulative wealth quintiles bracket) and the richer (individuals in the 50% - 75% cumulative wealth quintiles bracket). This means that, as individuals become rich and richer, they may change lifestyle relating to alcohol intake and may consume more alcohol. This is consistent with the studies of Hosseinpoor *et al*. [10] and Habib & Soma [6]. This increased alcohol consumption may perhaps be due to their ability to afford categories of alcoholic beverages, social networks interactions and frequently attending to social functions and programmes. In relation to wealth quintiles and tobacco use, the current study noted that, virtually, smoking is non-existent among the poor and the richest. This finding was contrary to the study outcome of Owusu-Dabo *et al*. [20] where increased smoking was found in poor and vulnerable communities. The study results revealed that, the few pockets of smoking identified were found among the rich and richer categories, denoting (25% - 75% cumulative wealth quintiles bracket).

Again, the concentration curve drawn above the line of perfect equality (45° angle) shows a positive concentration which seem to suggest that, the few people involved in smoking are addicts and may be deduced from the frequency of smoking, the number sticks they smoke per day or their use of tobacco in any form. The prevalence of smoking in Ghana may be low due to the implementation of the framework convention on tobacco control (FCTC) and the measures put forth by the Ghana Committee on Tobacco Control [21,22] such as the institution of no smoking in public places [23]. The study noted that, very marginal unhealthy dietary practice was found among the poor and rich (25% - 50% cumulative wealth quintiles bracket). Research outcomes have revealed that, the rich people are perceived to have poor dietary practices because, aside from the patronage of junk foods, they consumed protein and other minerals in excess which are injurious to health. However, a close look at the outcome of this study seem to tell the story that, there is virtually unhealthy dietary practice across all wealth quintiles in Ghana. For instance, studies have reported of malnutrition among the poor and vulnerable communities [24-26] and over nutrition, consumption of junk foods as well as untimely eating among the rich [12,27]. Lastly, the study found that, physical inactivity is almost concentrated among all the wealth quintiles with the incidence of concentration falling more on the rich and richer quintiles (25% - 75% cumulative wealth quintiles bracket). This may possibly be due to the fact that, the individuals within the rich and richer wealth categories are involved in white collar jobs, commercial activities and professional works which may involve fewer physical activities.

## Conclusion

Virtually, both poor and rich people are at risk in acquisition of non-communicable diseases due to unhealthy dietary practices such as inadequate fruits and vegetables intake, composition of balanced diet and sedentary lifestyles.

**Recommendations:** the study recommends that, information on healthy dietary practices should be made available to both the poor and rich. The implications of unhealthy dietary practices should be opened to the public by the community health promotion professionals. Even though some research outcomes have mentioned that NCDs are no longer the diseases of only the rich people, this information has not been disseminated well to the population. Effective education on NCDs occurrence cutting across the wealth quintiles should be brought to public domain. Disease conditions emerging out of risk factors of NCDs are multi-faceted and therefore it is recommended that, a multi-disciplinary body be established to put up preventive measures to reduce the magnitude of people affected by the risk factors of NCDs.

### What is known about this topic

- *The risk factors of non-communicable diseases are known;*
- *There is therefore the evidence that, social and economic status of a person and NCDs risk factors are associated*.

### What this study adds

- *Again, the study revealed that, wealth quintiles are practically indifferent to tobacco use and that both the rich and poor equally and minimally use tobacco as the concentration curve is very close to the perfect line of equality (45°);*
- *This study found near equal distribution of unhealthy dietary practices among the rich and poor in Ghana;*
- *It was found that, 40% - 80% of rich people were physically inactive with the application of a physical activity level of 100%*.
